# Identification of the IGF1/PI3K/NF *κ*B/ERK gene signalling networks associated with chemotherapy resistance and treatment response in high-grade serous epithelial ovarian cancer

**DOI:** 10.1186/1471-2407-13-549

**Published:** 2013-11-16

**Authors:** Madhuri Koti, Robert J Gooding, Paulo Nuin, Alexandria Haslehurst, Colleen Crane, Johanne Weberpals, Timothy Childs, Peter Bryson, Moyez Dharsee, Kenneth Evans, Harriet E Feilotter, Paul C Park, Jeremy A Squire

**Affiliations:** 1 Department of Pathology and Molecular Medicine, Queen’s University, Kingston, ON, Canada; 2 Department of Biomedical and Molecular Sciences, Queen’s University, Kingston, ON, Canada; 3 Department of Physics, Engineering Physics and Astronomy, Queen’s University, Kingston, ON, Canada; 4 Ontario Cancer Biomarker Network, Toronto, ON, Canada; 5 Department of Pathology, The Ottawa Hospital, Ottawa, ON, Canada; 6 Centre for Cancer Therapeutics, Ottawa Hospital Research Institute, Ottawa, ON, Canada; 7 Department of Obstetrics and Gynecology, Queen’s University, Kingston, ON, Canada; 8 Departments of Genetics and Pathology, Faculdade de Medicina de Ribeirão Preto, University of Sao Paulo, Brazil

**Keywords:** Ovarian cancer, Chemotherapy resistance, Biomarkers, Gene expression, Microarray

## Abstract

**Background:**

Resistance to platinum-based chemotherapy remains a major impediment in the treatment of serous epithelial ovarian cancer. The objective of this study was to use gene expression profiling to delineate major deregulated pathways and biomarkers associated with the development of intrinsic chemotherapy resistance upon exposure to standard first-line therapy for ovarian cancer.

**Methods:**

The study cohort comprised 28 patients divided into two groups based on their varying sensitivity to first-line chemotherapy using progression free survival (PFS) as a surrogate of response. All 28 patients had advanced stage, high-grade serous ovarian cancer, and were treated with standard platinum-based chemotherapy. Twelve patient tumours demonstrating relative resistance to platinum chemotherapy corresponding to shorter PFS (< eight months) were compared to sixteen tumours from platinum-sensitive patients (PFS > eighteen months). Whole transcriptome profiling was performed using an Affymetrix high-resolution microarray platform to permit global comparisons of gene expression profiles between tumours from the resistant group and the sensitive group.

**Results:**

Microarray data analysis revealed a set of 204 discriminating genes possessing expression levels which could influence differential chemotherapy response between the two groups. Robust statistical testing was then performed which eliminated a dependence on the normalization algorithm employed, producing a restricted list of differentially regulated genes, and which found *IGF1* to be the most strongly differentially expressed gene. Pathway analysis, based on the list of 204 genes, revealed enrichment in genes primarily involved in the IGF1/PI3K/NF *κ*B/ERK gene signalling networks.

**Conclusions:**

This study has identified pathway specific prognostic biomarkers possibly underlying a differential chemotherapy response in patients undergoing standard platinum-based treatment of serous epithelial ovarian cancer. In addition, our results provide a pathway context for further experimental validations, and the findings are a significant step towards future therapeutic interventions.

## Background

Ovarian cancer remains the most common cause of death in women due to a gynecological malignancy [1]. Unfortunately, most women first present with advanced disease. According to the Federation of Obstetricians and Gynecologists (FIGO) international system, Stage I ovarian cancer is identified as a tumour that is restricted to the ovaries. The cancer is defined to be Stage II when both ovaries are involved and the tumour has extended to the pelvis. Stage III and IV are identified when the tumour shows peritoneal metastasis and distant metastasis, respectively. Given the absence of an effective screening test and the lack of specific symptoms, the majority of women present with stage III or IV disease. The standard frontline therapy for advanced ovarian cancer is debulking surgery and platinum-paclitaxel based combination chemotherapy. Despite major advances in the development of novel treatment regimens and targeted therapies, such as immunotherapy, cytotoxic and anti-angiogenic therapies, there has been only a marginal improvement in the survival of women with ovarian cancer over recent decades, largely due to refinements in chemotherapy and surgical technique [2]. However, recent literature suggests a more refined understanding of the biological mechanisms underlying this disease [3]. Molecular classifications have been used to broadly divide ovarian cancer as Type I (mutations in *KRAS* and *BRAF* leading to activation of the MAPK pathway) or as Type II (extensive *TP53* mutations, and sometimes over expression of HER2/neu and AKT2) [4,5] tumours. In addition, it has been proposed that the molecular comparisons within individual histologic groups are more meaningful, as these subtypes are now considered to be different diseases that share the same anatomical site of growth [6].

Chemotherapy resistance is the major obstacle in treating women with ovarian cancer [7]. Based on the progression-free survival (PFS) after completion of chemotherapy, patients are classified as platinum-sensitive (PFS > eighteen months) or platinum-resistant (PFS < six months) [8]. Those women who progress between 6-12 months post treatment are considered to have tumours with reduced sensitivity to platinum. The percentage of complete and partial response is 75% in patients with the platinum-sensitive disease, but only 10-20% in the platinum-resistant disease [9]. The intermediate partially sensitive (or partially resistant) population has approximately a 30% chance of response to further platinum-based therapy [9]. Resistance to platinum-based chemotherapy is multifactorial, and exhibited either intrinsically or acquired with drug exposure. It is thought that there may be pre-existing resistance mutations in tumours prior to treatment, thus accounting for the high frequency of platinum-resistant ovarian cancer at first relapse [8]. In addition, an active interaction between the drug and tumour microenvironment may lead to selective up or down-regulation of genes involved in the pathways associated with a variation in response to chemotherapy [10]. The major advantage of identifying pathways involved in intrinsic chemotherapy resistance is that targeted strategies can be developed for an earlier time point in the disease process to address the cellular responses that become activated upon drug exposure [11].

There have been various studies in recent years attempting to investigate associations between gene expression profiles in ovarian cancer and resistance to chemotherapy [12-17]. Whilst these studies have addressed differential gene expression with various clinical correlates, many have included a range of histologies or uniquely cell line data [18-20]. The objective of the present study was to use gene expression profiling of a carefully selected group of patients distinguished predominantly by their varying responses to chemotherapy, using progression free survival (PFS) time as a surrogate of drug response. This group of patients was considered homogeneous with respect to all other clinical features apart from PFS. The selected 28 serous epithelial ovarian cancer (SEOC) tumours comprised a discovery cohort that could be used to identify key molecular networks associated with intrinsic chemotherapy resistance in SEOC patients receiving standard treatment. Robust statistical analyses were used to define a set of distinguishing genes that were used for pathway analysis. This list of genes could be used to validate potential biomarkers in other cohorts that are involved in a differential response to chemotherapy in SEOC.

## Methods

### Ethics statement

Institutional ethics approval was obtained from Queen’s University and the Ottawa Hospital Research Institute’s (OHRI) Research Ethics Boards. Informed written consent was obtained in all patients prior to sample collection.

### Patient tissue samples and classification

**Figure 1:**
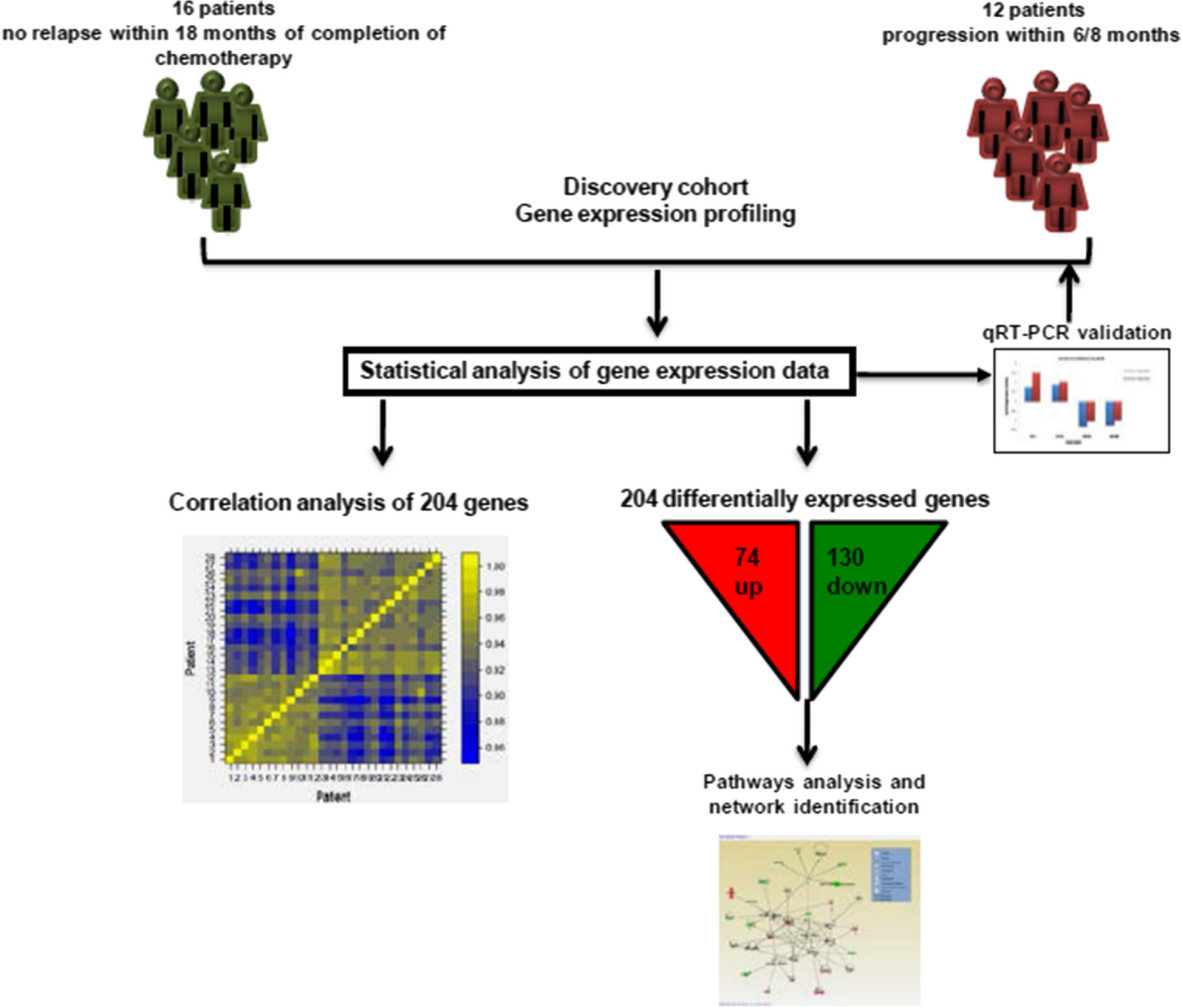
**Study Design.** A schematic representing the workflow followed when comparing and interpretting gene expression differences of 28 high-grade SEOC tumour samples that had been characterized using their progression-free survival. After sample characterization, gene expression intensities were analyzed, correlations between patients for the 204 differentially regulated genes were quantified, and microarray gene expression were validated with qRT-PCR. Ingenuity Pathway Analysis was completed with the fold change values for the 204 differentially regulated genes, and the networks having the highest scores were studied.

A cohort of 28 locally advanced (IIa-IV) fresh frozen high-grade SEOC tumours were obtained from the Ontario Tumour Bank and the OHRI. Tumour samples were collected at the time of primary debulking surgery, and stored at -80°C until processing. Patients were naive to chemotherapy and radiotherapy prior to cytoreductive surgery and standard carboplatin/paclitaxel chemotherapy. Histological classification of the tumours was performed using the WHO criteria, and disease staging according to the International Federation of Gynecology and Obstetrics (FIGO) guidelines. Histopathological examination of the tumour sections performed by a pathologist (TC) confirmed more than 70% tumour in all samples. As per the Gynecologic Cancer Intergroup Guidelines, patients were classified into two arms using either Ca-125 or RECIST criteria, and were assigned to either the sensitive or the partially resistant/resistant groups based on their PFS (Table 1). Two distinct arms were selected for study based on their clear separation according to their respective PFS. Twelve samples were classified as partially resistant/resistant, as they exhibited progressive disease within eight months from completion of chemotherapy. In contrast, sixteen samples demonstrated high sensitivity to platinum, as there was no relapse within 18 months after completion of chemotherapy. A schematic representation of the overall study design is presented in Figure 1.

**Table 1:** Clinical and pathological characteristics of adjuvant treated SEOC patient samples

**Sample**	**Age**	**Stage**	**PFS (months)**	**Classification**
1100	54	IIIc	5	Resistant
1101	55	IV	5	Resistant
1240	64	III	<3	Resistant
1299	53	IIIb	3	Resistant
1413	47	IV	<3	Resistant
1587	54	IIIc	0	Resistant
1605	51	IIb	3	Resistant
1703	48	IIIc	6	Resistant
1776	58	IIIc	6	Resistant
D00443	51	III	5	Resistant
1359	62	IIIc	8	Partially Resistant
1680	67	IIc	7	Partially Resistant
1157	61	IIIc	36	Sensitive
1188	58	III	34	Sensitive
1224	–	–	22	Sensitive
1296	61	II	39	Sensitive
1304	51	IIIa	26	Sensitive
1308	49	IIIc	25	Sensitive
1351	55	IV	No recurrence	Sensitive
1355	68	IIa	23	Sensitive
1381	51	IIIa	25	Sensitive
1561	63	IIb	25	Sensitive
1625	54	IIIb	26	Sensitive
1627	52	IIIa	No recurrence	Sensitive
1706	61	IIIa	No recurrence	Sensitive
B01183	79	IIIc	22	Sensitive
B01360	54	IIIc	19	Sensitive
B01440	53	IIIc	20	Sensitive

### Gene expression profiling

Total RNA was isolated from all tumour samples using a combination of Trizol (Invitrogen, CA) and Qiagen RNA isolation kit (Qiagen Inc., Mississauga, CA), as per manufacturer’s instructions. The RNA integrity was analyzed using RNA 6000 Nano Chip on an Agilent 2100 Bioanalyzer (Agilent Technologies, USA). The RNA concentration was determined spectrophotometrically on a NanoDrop ND-100 spectrophotometer (NanoDrop Technologies, USA). All samples showed appropriate RNA integrity number, and were thus subjected to downstream microarray analysis. All the hybridization experiments were performed using Affymetrix Human Genome U133 Plus 2.0 arrays (Affymetrix Inc., USA) at the Centre for Applied Genomics (The Hospital for Sick Children, Toronto, ON, Canada). 500 nanograms of total RNA was used for cDNA synthesis using GeneChip 3 ^′^ IVT Express Kit (Affymetrix Inc, Santa Clara, CA USA). Post hybridization array washing, scanning and probe quantification was performed on an AffymetrixGeneChip Scanner 3000, as per manufacturer instructions. The gene expression raw data files have been deposited to NCBI Gene Expression Omnibus (GEO accession GSE51373 at http://www.ncbi.nlm.nih.gov/projects/geo/).

### Microarray data analysis

The normalization of the microarray data was conducted using packages available in R/Bioconductor. Significance tests and other analysis was completed using standard statistical functions in R.

Technical microarray quality control analysis was performed on the full set of CEL files using the *arrayQualityMetrics* Bioconductor package, based on the 12 samples from the resistant cohort, and 16 samples from the sensitive cohort [21]. Normalization was performed over all 28 samples and all 54,675 probe sets using the MAS5 algorithm from the *affy* Bioconductor package [22]. This normalization processing was chosen for a variety of reasons. First, although it is recognized that different normalizations tend to give different answers [23], thereby leading to different conclusions, it has been suggested that MAS5 is appropriate for identifying differences between various sets of data. Indeed, in comparison to other normalization methods we obtained the largest number of differentially regulated genes when the MAS5 normalization was used. Second, when a variety of normalizations were employed, specifically the four normalization algorithms MAS5, LiWong [24], RMA [22] and gc-RMA [25], the MAS5 values were, in fact, closest to the averages obtained from taking the mean expression intensity of the four normalization results. Finally, from the MAS5 expression intensities, the log_2_ value of the mean expression intensity of the resistant cohort relative to the mean expression intensity of the sensitive cohort was calculated.

### Quantitative reverse transcriptase PCR (qRT-PCR) analysis

Gene expression changes as calculated using the comparative *ΔΔC*_*t*_ method [26] were obtained from qRT-PCR studies for technical validation. For this experiment, qRT-PCR was performed in all 28 samples in triplicate. Two over-expressed (*IGF1* and *ZFP36*) and two under-expressed genes (*ZNF83* and *MCM8*) were examined, and their expression differences were obtained relative to the house-keeping control gene *ACTB*.

### *In silico* validation of gene expression analysis

We performed *in silico* validation of our gene expression profiling results using data from The Cancer Genome Atlas (TCGA). The TCGA dataset contains microarray based gene expression data from over 500 high-grade ovarian cancer samples. We selected 19 resistant and 25 sensitive samples for a comparative validation study. The selection of these two groups from the TCGA dataset was based on similar clinical criteria as applied to our discovery cohort. With these 44 samples we completed the same MAS5 normalization gene expression differentiation analysis as described above for the discovery cohort of 28 samples.

## Results and discussion

### Gene expression analysis

The process of identifying probe set expression intensities corresponding to significantly different expression intensity averages is somewhat complicated by the fact that for the small sample numbers, twelve resistant and sixteen sensitive, the distributions of expression intensities is not normal. In our expression dataset we often find bimodal, multimodal, or uniform distributions, which is simply a bi-product of working with small sample numbers, as is often found. Therefore, in addition to performing a Welch two-sample *t* test, corresponding to a parametric procedure, we also examined the expression intensities for all probe sets using the non-parametric Mann-Whitney *U* test procedure. Following this approach, a probe set was identified to possess a significantly different expression intensity distribution for the resistant and sensitive cohorts if (i) the *p* value for each test was less than 0.01, and (ii) the absolute value of the log_2_ fold change was in excess of 0.2. The Welch procedure generated a list of 434 probe sets, and the Mann-Whitney procedure then reduced this to a collection of 310 probe sets. Due to our use of multiple significance tests, no corrections using a chosen false discovery rate were performed.

To obtain a list of differentially expressed genes, from the collection of 310 probe sets, the probe sets that were not identified with a gene, the open reading frame and hypothetical genes were all ignored. Our final analysis was based on this reduced list of 219 probe sets. From this list of 219 probe sets one finds a small number of duplicated genes, so-called redundant expression levels. A cluster “averaging” over probe sets consistent with the SCOREM algorithm, recently proposed to handle such redundant probe sets [27], was used. Therefore, at the conclusion of this statistical processing our analysis produces a list of 204 genes, and when ordered by their log_2_ fold change values these are given in the Additional file 1 available with this report (Additional file 1: Table S1). It is noteworthy that 74 probe sets had higher expression values for the resistant cohort versus the sensitive cohort, whereas the 130 had lower expression levels for the resistant cohort. Therefore, on average the differentially regulated genes that distinguish the two cohorts are more likely to be underexpressed in the resistant tumours than in the sensitive tumours, suggesting that loss or reduced expression of key genes may underlie varying cellular responses to chemotherapy.

The potential caveat to the above results, as mentioned, is that different normalizations lead to variable subsets of differentially expressed genes. To circumvent the potential bias introduced by choosing one normalization method (MAS5), further analysis was taken in which a probe set was identified to possess a significantly different expression intensity distribution for the resistant and sensitive cohorts if (i) the *p* value for both tests (parametric and non-parametric) was less than 0.01, and (ii) the absolute value of the log_2_ fold change was in excess of 0.5, and (iii) the probe set must be identified for all four normalizations considered. The resulting robust list of 32 differentially expressed genes contained genes with (absolute) log_2_ fold changes between 0.5 and around one, except for one gene, *IGF1*. When averaged over the four normalizations, *IGF1* is found to have an average fold change of 1.6 ±0.2.

### Correlations

**Figure 2:**
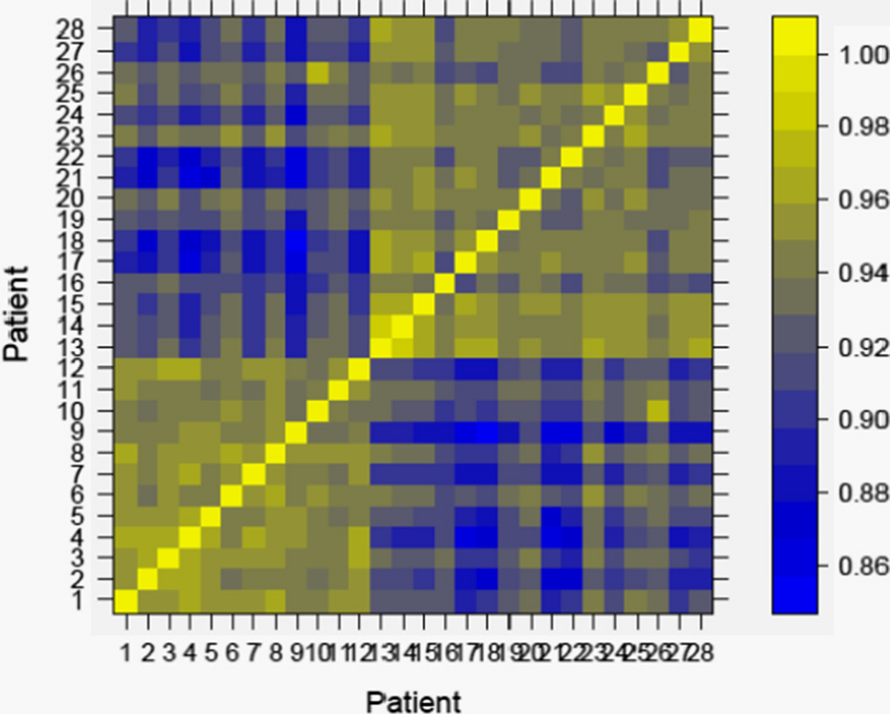
**Correlation Map.** The Spearman rank correlation coefficient rho for the supervised list of differentially expressed genes, evaluated between all different pairs of tumour samples. The results are plotted using the levelplot function of R. The colour legend on the right hand side of the figure indicates that bright yellow corresponds to a value of rho nearly equal to one, whereas bright blue is assigned to values of rho close to 0.85. No values of rho less than 0.85 are obtained. The resistant patients are given patient identifiers between 1 and 12, whereas the sensitive patients are given patient identifiers between13 and 28. This pair-wise display of all 28 samples clearly shows the similarity in expression profiles of all tumours within the 12 tumour resistant group, which can clearly be distinguished from the similarities of expression of all tumours within the 16 tumour sensitive group. The high degree of homogeneity within each of these two groups, and the dissimilarities between the resistant and sensitive tumour groups, provides strong evidence for the robustness of the identification and statistical evaluation of the 204 differentially expressed genes.

To better appreciate the degree of similarity and dissimilarity of gene expression intensities of all 204 genes across the entire cohort of 28 tumours, we performed an inter-sample correlation analysis - similar ideas have appeared in published gene expression papers [28]. The most differentially expressed 204 genes that distinguish between the chemo-resistant and chemo-sensitive cohorts, described above, are given in Additional file 1: Table S1. The gene expression intensities of each patient were then ranked, and the inter-patient Spearman rank correlation coefficient, *ρ*, was evaluated [29]. Our results are shown in Figure 2. A value of *ρ* close to one indicates a monotonically changing relationship between the supervised gene list of pairs of patient tumours, and no *ρ* values less than 0.85 are found. This pair-wise display of all 28 samples clearly shows the similarity in expression profiles of all tumours within the 12 tumour resistant group, which can clearly be distinguished from the similarities of expression of all tumours within the 16 tumour sensitive group. The high degree of homogeneity within each of these two groups, and the dissimilarities between the resistant and sensitive tumour groups, provides strong evidence for the robustness of the identification and statistical evaluation of the 204 differentially expressed genes. The correlation analysis also confirms that the rationale for the initial selection of the two tumour groups based on each patient’s PFS as a surrogate of their chemotherapy response was appropriate.

### Technical validation of microarray results

**Figure 3:**
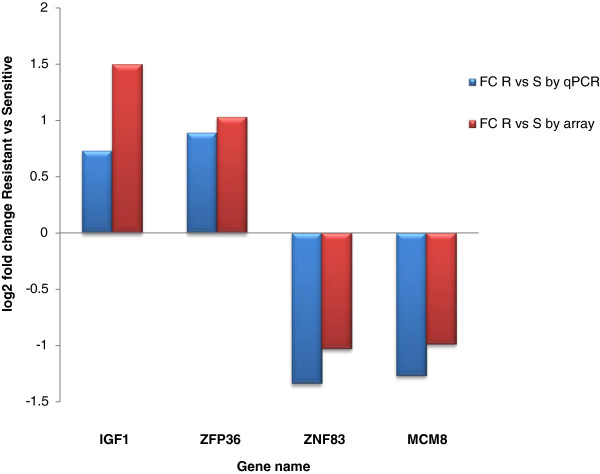
**qRT-PCR Validation Results.** A comparison of the qRT-PCR data to the microarray data, the latter obtained from the MAS5 normalization algorithm, for a set of two over-expressed and two under-expressed genes. The fold change refers to a ratio of the resistant to sensitive mean expression intensities.

Two over-expressed (*IGF1* and *ZFP36*) and two under-expressed (*MCM8* and *ZNF83*) genes that were significantly differentially expressed were analyzed on all 28 samples by qRT-PCR. Our results, compared to the microarray log_2_ fold changes for these same genes when analyzed using the MAS5 normalization, are shown in Figure 3. From these results one sees that the expression differences detected on the microarrays were also evident using other measures of assessing expression levels. These data also confirmed the directionality of the fold change differences as revealed by microarray analysis.

### Gene signatures and major signalling pathways associated with chemotherapy resistance

**Figure 4:**
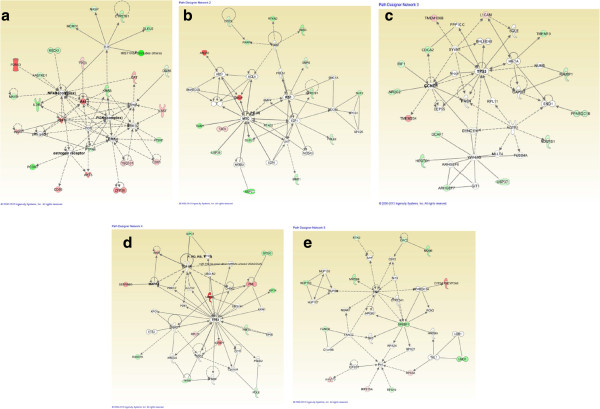
**Gene networks generated by Ingenuity Pathway Analysis of 204 differentially expressed genes.a**. Highest scoring signalling network 1 showing the IGF1, PI3K/Akt, NFkB and ERK signalling axes. Molecular relationships with genes from the current study (highlighted in green/red), as well as from the IPA knowledge base, are shown. Green indicates over-expressed whereas red indicates under-expressed genes. Other signalling pathways such as E2f, Vegf and estrogen receptor are also seen. Associated functions include cellular development, cellular growth and proliferation, and cell cycle. **b**. Signalling network 2 showing the MYC and RB1 signalling axis. Molecular relationships with genes from the current study (highlighted in green/red), as well as from the IPA knowledge base, are shown. Associated functions include cell cycle, connective tissue development and function, cell death and survival. **c**. Signalling network 3 showing the TP53 and CCND1 signalling axis. Associated functions include cell morphology, cellular assembly and organization, and cellular development. **d**. Signalling network 4 showing the IGF1R, MAPK8 and TP53 complex signalling axes. Associated functions include cell cycle, cell death and survival, and cellular development. **e**. Signalling network 5 showing the TNF and SREBF1 complex signalling pathways. Associated functions include lipid metabolism, molecular transport, and small molecule biochemistry.

Ingenuity pathway analysis (IPA) was performed on the set of 204 differentially expressed genes, including their fold change values, in order to identify the most significantly altered gene networks, and the associated functions distinguishing the two groups. IPA employs Fisher’s exact test to determine the relationship between the input dataset and the canonical pathways with associated biofunctions. Molecular interaction networks explored by IPA tools, with the threshold settings of a maximum 35 nodes per network, revealed a total of 25 networks. The top five significant networks, containing at least thirteen differentially regulated genes in each network from the current data set, are shown in Figures 4a-e. Network 1 included 25 differentially regulated genes with signalling in IGF1, the NF *κ*B complex, PI3K, Akt, and ERK as the major over-represented gene networks. The high degree of relevance of these networks as potential drivers of PFS and drug response is reflected by the high proportion of genes from our 204 gene set being involved in each of the signalling networks. For example, 26 out of the 35 genes in network 1 were derived from the 204 gene set. Network 2 included 17 genes from the set and these genes are associated with MYC and RB1 signalling pathways. Similarly, the networks 3, 4 and 5 consisted of 14, 13 and 13 genes from the dataset. The major over-represented signalling networks associated with these networks were CCND1, TP53, IGF1R, and TNF. Cellular movement, growth and proliferation, DNA replication, recombination and repair, cell-to-cell signalling and cellular development were the predominant biological functions associated with the top five networks.

What is notable about these results is that the IPA analysis was completed using the 204 genes found from the MAS5 normalization. The network with the highest score, 41 in comparison to a score of 23 for the second highest scoring network, involves the *IGF1* gene. It is the same gene which was identified as possessing the most differentially expressed intensity when a normalization-independent significance analysis was completed, producing a robust list of differentially regulated genes. The appearance of this gene in multiple analyses highlights its putative role in understanding the biology of the chemo-resistant cohort.

### *In silico* validation of microarray results

We performed *in silico* validation of our microarray results, using data from TCGA ovarian cancer cohort, with the analysis parameters identical to our discovery cohort. The platform used for the TCGA analysis was Affymetrix U133, which has a different coverage than the platform we used for our discovery cohort (Affymetrix U133 Plus 2.0). The TCGA data analysis lead to the identification of an entirely distinct differentially expressed gene list (Additional file 2) compared to our discovery cohort. However, interestingly, when we subjected the differential gene list derived from this TCGA comparison study, to pathway analysis using the same parameters, we noted NF *κ*B, IGF1-R and ERK gene signalling networks in the top two networks.

## Conclusions

The present study was aimed at identifying gene expression markers of intrinsic chemotherapy resistance in high-grade SEOC patients. Chemotherapy-naive tumour samples from late stage, high-grade SEOC were selected to compare two distinct drug sensitivity profiles within this cohort of 28 patients, using comparative gene expression profiling by a high resolution Affymetrix gene expression microarray platform. The study was designed to identify the genes whose overall expression levels were discriminating between the twelve resistant/partially resistant patients and the sixteen chemotherapy sensitive patients selected for each cohort. Gene expression analysis in these two highly homogeneous groups of patients indicates the potential role of IGF1 as one of the key signalling pathways involved in the development of intrinsic chemotherapy resistance in ovarian cancer.

Insulin-like growth factor is produced by different cell types, and its role in cancer is well documented in prostate cancer, breast cancer, colorectal cancer and melanoma, where increased risks to these cancers were associated with higher IGF1 levels [30-35]. Also, the potential role of IGF1, along with IGFBP3, as prognostic markers that can predict mortality in men with advanced prostate cancer, was reported in a recent clinical study [36]. The activation of oncogenic “ *β*-catenin signalling through the inactivation of glycogen synthase kinase 3” (GSK3”) has also been shown to be associated with cancer stemness and chemo-resistance [37,38]. Recent studies suggest that the mechanisms of carcinogenesis and chemo-resistance exhibited by cancer cells are often due to the expression of the IGF1 receptor [39,40]. Drugs, including antibodies, targeting the insulin-like peptides signalling through the PI3K/Akt/mTOR pathway are currently in various clinical trials in breast and prostate cancers [41-44].

Previous studies on the role of IGF1 in ovarian cancer show that elevated serum levels of IGF1 are often observed in this cancer [45]. Higher levels of IGF1 are also found to be associated with increased disease risk, tumour metastasis and poor prognosis in ovarian cancer [46] via the activation of IGF1-R. A recent *in vitro* study indicated the role of IGF1 in enhancing ovarian cancer cell proliferation through PI3K/Akt/mTOR signalling [47]. Exogenous addition of IGF1 in ovarian cells also leads to their increased proliferation [48]. *In vitro* findings indicate the role of IGF1-R and PI3K in cisplatin resistance [49]. Based on earlier findings on the role of IGF1 in low-grade ovarian carcinomas [46], as well as in *in vitro* studies in hepatocellular carcinoma, a phase II clinical trial is currently underway using the IGF-1R/IR dual receptor tyrosine kinase inhibitor OSI-906 (clinicaltrials.gov). However, the role of IGF1 in the development of chemo-resistance in ovarian cancer has not yet been defined in patient cohorts that exhibit resistance to chemotherapy. It has been reported that a compensatory mechanism imparted by one receptor tyrosine kinase for another eventually leads to drug resistance in targeted therapies [50]. Zhao and colleagues [51] report a strong correlation between EMT status and sensitivity to IGF1-R/IR inhibitor OSI-906. Our current findings on relatively increased expression of *IGF1* in the resistant patients indicate that gene expression based predictive biomarkers in this pathway might be considered for future clinical trials. The relative increased expression of *INSR* (a receptor for insulin) and *IGF1* in the resistant cohort in our study indicates that the drug resistant cells evolve multiple compensatory mechanisms for tumour cell survival. Our study, therefore, also confirms the *in vitro* findings at the clinical level, where the deregulated IGF1 pathway might play a role in intrinsic chemotherapy resistance.

The genes in the PI3K/Akt cascade were recently shown to induce drug resistance to cisplatin in vitro using an integrative gene expression and pathway based approach [52]. Activation of the PI3K pathway involves alterations in any of the downstream or upstream molecules involved along the PI3K/Akt/mTOR axis. This knowledge has not yet been translated into the use of targeted therapies in the treatment of ovarian cancer, and further studies are needed to improve our understanding of the molecular pathways that govern chemotherapy response in SEOC. The PI3K pathway is activated by a number of growth factors including *IGF1*, resulting in cellular growth and metastasis as well as chemotherapy resistance. Blocking the PI3K/Akt pathways both *in vitro* and *in vivo* has been shown to increase drug efficacy in controlling tumour cell growth and proliferation [53].

Our *in silico* validation of gene expression results using a subset of the TCGA data did not demonstrate overlap between the 204 gene list (Additional file 1: Table S2) and TCGA gene list of 109 genes (Additional file 1: Table S2). In light of the high level of genomic diversity recently identified in untreated high-grade SEOC tumours [54], it is not surprising that there is considerable variability at the expression level of individual genes. However, when the TCGA gene set of 109 differentially expressed genes was subjected to IPA analysis, ERK and NF *κ*B and IGF1-R networks appeared in the top two networks. This finding suggests that pathway alterations are likely more important *per se* than the identity of the actual genes that lead to dysregulation of expression [17]. Several different independent gene expression profiling studies have led to the discovery of different sets of genes lists [10,55-57]. However, the major pathways that are consistently associated with chemotherapy resistance in ovarian cancer remain the same. In addition to IGF1, pathway analysis in our study also identified NF *κ*B and ERK signalling as the major overrepresented networks in the resistant group compared to the sensitive. This finding is consistent with a recent study based on the publicly available TCGA dataset, which reports the overrepresentation of NF *κ*B and ERK signalling based on IPA analysis of differential gene sets [58]. A previously reported study, using gene expression profiling, conducted to delineate intrinsic chemotherapy resistance pathways, showed an involvement of cell-cycle, extracellular matrix, cell adhesion and signalling associated genes in the chemotherapy resistant group [22]. Earlier reports also indicate the role of cell cycle regulators such as cyclins in response to treatment with platinum-based therapies [59]. Another study identified a 320-gene set that distinguishes the chemotherapy sensitive tumours [56]. Up-regulation of genes involved in cell cycle regulation, down-regulation of genes involved in cell adhesion, transcriptional regulation and signal transduction was also reported [56]. However, overall previous studies indicate a role of genes involved in cell cycle regulation, cell adhesion and signal transduction in the development of a chemotherapy resistance, which is consistent with the findings in our study.

One of the major findings of our study is the role of IGF1 signalling in mediating intrinsic chemotherapy resistance, possibly by activation of the PI3K/Akt, NF *κ*B and ERK pathways. Since increased NF *κ*B activation also correlates with chemotherapy resistance in solid tumours [60], it could be argued that drug resistant cells reside within the tumour and exhibit inherent activation of multiple signalling pathways, which eventually lead to tumour recurrence. In addition, given that IGF1 can activate the PI3K as well as the ERK signalling pathway, it might be possible that increased NF *κ*B activation is initiated as a result of increased levels of IGF1 in the resistant population. These cells might further contribute to the survival, proliferation and recurrence following chemotherapy. As described in the results, the *IGF1* gene emerged from both pathway analysis (network 1), and as the highest differentially expressed gene in the robust list generated by the application of four different normalization methods. This emphasizes the potential role of IGF1 in PFS, and potentially in intrinsic chemotherapy resistance.

The differential expression of the 204 gene set when the two groups were compared provides experimental evidence of major signalling pathways leading to difference in PFS associated with the development of the chemotherapy resistant phenotype. Our results support that, in addition to the classical drug resistance pathways, other major gene networks may interact by various mechanisms to confer differential response to chemotherapy. The current study highlights the role of the intrinsic ability of cancer cells to respond to a drug-resistant phenotype which, upon exposure to combination chemotherapy, may initiate a cascade of complex pathway activations leading to drug resistance.

## Abbreviations

SEOC: Serous epithelial ovarian cancer; PFS: Progression free survival.

## Competing interests

The authors declare that they have no competing or financial interests.

## Authors’ contributions

MK carried out sample processing, subjected samples to microarray processing, and wrote the manuscript with RG and JS. JW carried out classification of primary tumours as chemotherapy sensitive or resistant using clinical data. CC helped with sample acquisition. TC performed the histopathological analysis of the FFPE sections. PN performed array quality metrics analysis. RG performed the microarray data analysis. JS, PP and HF, KE, MD, PB conceived the study. All authors read and approved the final manuscript.

## Pre-publication history

The pre-publication history for this paper can be accessed here:

http://www.biomedcentral.com/1471-2407/13/549/prepub

## Supplementary Material

Additional file 1 **List of Differentially Regulated Genes.** As described in Methods, using the MAS5 normalization algorithm a list of differentially regulated genes was created. These genes have been found to have mean expression intensities that are significantly different, when the tumour samples were grouped into the resistant and sensitive cohorts. Genes that are coloured blue are redundant genes for which multiple probe sets of the microarray were found to be differentially expressed. Click here for file

Additional file 2 **Differentially expressed genes in a resistant cohort compared to a sensitive cohort.** The gene list was derived from an independent in silico validation of gene expression analysis using TCGA ovarian cancer data sets (19 sensitive and 25 resistant samples) with identical data analysis parameters as applied for the discovery cohort. Click here for file
