# Supplementary material for: Identification of the IGF1/PI3K/NF κB/ERK gene signalling networks associated with chemotherapy resistance and treatment response in high-grade serous epithelial ovarian cancer
Source: BMC Cancer. 2013 Nov 16;13:549. doi: 10.1186/1471-2407-13-549 (PMC3840597; doi:10.1186/1471-2407-13-549)
Supplement: Additional file 1 — List of Differentially Regulated Genes. As described in Methods, using the MAS5 normalization algorithm a list of differentially regulated genes was created. These genes have been found to have mean expression intensities that are significantly different, when the tumour samples were grouped into the resistant and sensitive cohorts. Genes that are coloured blue are redundant genes for which multiple probe sets of the microarray were found to be differentially expressed. [file 1471-2407-13-549-S1.pdf]

Gene Symbol	Chromosomal Location	log2(FoldChange)
RBM20	chr10q25.2	2.20
UBE3A	chr15q11.2	2.12
ATRNL1	chr10q26	2.02
NCRNA00161	chr21q21.3	1.87
ABCB5	chr7p21.1	1.85
MGP	chr12p12.3	1.71
P2RX3	chr11q12	1.67
CORO2B	chr15q23	1.63
CCDC83	chr11q14.1-q14.2	1.57
DENND2A	chr7q34	1.55
MYT1L	chr2p25.3	1.53
IGF1	chr12q23.2	1.50
EPHA5	chr4q13.1	1.33
LEPREL1	chr3q28	1.30
GPR156	chr3q13.33	1.30
AKT3	chr1q43-q44	1.29
DKK3	chr11p15.2	1.25
CYP2A6	chr19q13.2	1.19
DSCAM	chr21q22.2	1.16
IGFBP7	chr4q12	1.10
RGS8 / SDHAP3	chr1q25 / chr5p15.33	1.08
ABCA9	chr17q24.2	1.06
SPON2	chr4p16.3	1.06
ZNF518B	chr4p16.1	1.05
SLC24A5	chr15q21.1	1.05
ZFP36	chr19q13.1	1.03
PML	chr15q22	1.00
FAM126A	chr7p15.3	0.98
SERPINB3	chr18q21.3	0.96
ANTXR2	chr4q21.21	0.95
TMEM204	chr16p13.3	0.94
PGCP	chr8q22.2	0.93
GTF2H2B	chr5q13.2	0.89
STX2	chr12q24.33	0.89
CD59	chr11p13	0.82
SPTBN4	chr19q13.13	0.82
SNX3	chr6q21	0.81
CAT	chr11p13	0.81
MIR770	chr14q32.2	0.79
INSR	chr19p13.3-p13.2	0.75
VKORC1	chr16p11.2	0.75

IGHV3OR16-14	chr16p11.2	0.72
EIF3F	chr11p15.4	0.70
IL6ST	chr5q11	0.67
MOSPD1	chrXq26.3	0.64
RNF133	chr7q31.32	0.64
IPO11	chr5q12.1	0.60
SUMF2	chr7q11.1	0.60
WRB	chr21q22.3	0.60
TMEM106B	chr7p21.3	0.59
PPM1F	chr22q11.22	0.58
MMGT1	chrXq26.3	0.58
PCNX	chr14q24.2	0.57
RNH1	chr11p15.5	0.56
MICAL3	chr22q11.21	0.56
TPP1	chr11p15	0.55
RBMX	chrXq26.3	0.54
BTF3	chr5q13.2	0.54
GNA12	chr7p22.2	0.54
ZXDA / ZXDB	chrXp11.1 / chrXp11.21	0.53
METTL9	chr16p13-p12	0.53
CD81	chr11p15.5	0.52
TTC3	chr21q22.2	0.49
RPL10A	chr6p21.31	0.49
TAF9	chr5q11.2-q13.1	0.48
TSG101	chr11p15	0.47
SLC9A6	chrXq26.3	0.46
DNASE1L1	chrXq28	0.44
POFUT2	chr21q22.3	0.41
RPL23	chr17q	0.41
EIF1	chr17q21.2	0.39
L1CAM	chrXq28	0.39
RPS3A	chr4q31.2-q31.3	0.35
RPS15A	chr16p	0.25
PSMG2	chr18p11.21	-0.28
RAB35	chr12q24.31	-0.31
RNF10	chr12q24.31	-0.33
STARD7	chr2q11.2	-0.35
HMGXB3	chr5q32	-0.35
SNRNP200	chr2q11.2	-0.36
SP3	chr2q31	-0.36
ZNF131	chr5p12	-0.40
PHIP	chr6q14	-0.40
CRIPT	chr2p21	-0.41

YIPF1	chr1p33-p32.1	-0.43
DDX5	chr17q21	-0.44
KIAA1731	chr11q21	-0.45
RTN3	chr11q13	-0.45
TRIT1	chr1p34.2	-0.46
KIAA0467	chr1p34.2	-0.49
NASP	chr1p34.1	-0.49
RPS19	chr19q13.2	-0.49
RPS6KB2	chr11q13.2	-0.50
AFG3L2	chr18p11	-0.51
RREB1	chr6p25	-0.51
GMPS	chr3q24	-0.51
CEP192	chr18p11.21	-0.52
TESK1	chr9p13	-0.52
CHMP1B	chr18p11.21	-0.53
RIF1	chr2q23.3	-0.54
ATAD2	chr8q24.13	-0.54
NSFL1C	chr20p13	-0.55
NDUFS1	chr2q33-q34	-0.55
PTPN6	chr12p13	-0.56
ORC2	chr2q33	-0.57
RBM26	chr13q31.1	-0.57
POLA2	chr11q13.1	-0.58
POLE	chr12q24.3	-0.59
SASS6	chr1p21.2	-0.59
RSF1	chr11q14.1	-0.59
FANCB	chrXp22.2	-0.59
DTX3L	chr3q21.1	-0.59
MRPS15	chr1p34.3	-0.60
IDH3B	chr20p13	-0.60
ARHGEF7	chr13q34	-0.61
DIS3L2	chr2q37.1	-0.61
FASTKD1	chr2q31	-0.62
RNF138	chr18q12.1	-0.62
RALBP1	chr18p11.3	-0.63
PPARGC1B	chr5q32	-0.63
USP37	chr2q35	-0.63
HINT1	chr5q31.2	-0.64
USP28	chr11q23	-0.64
TRMT6	chr20p12.3	-0.65
PRPF38B	chr1p13.3	-0.65
March7th	chr2q24.2	-0.65
ZBED6	chr1q32.1	-0.65

FAM111A	chr11q12.1	-0.65
USP15	chr12q14	-0.66
NAA15	chr4q31.1	-0.66
DCAF7	chr17q23.3	-0.67
ZCCHC3	chr20p13-p12.2	-0.68
PPP1R3E	chr14q11.2	-0.69
MTF2	chr1p22.1	-0.70
IL20RB	chr3q22.3	-0.70
NR2C2	chr3p25	-0.70
FAM72A / FAM72B / FAM72C / FAM72D	chr1p11.2 / chr1q21.1 / chr1q32.1	-0.72
THRB	chr3p24.2	-0.72
STK35	chr20p13	-0.72
TTF2	chr1p22	-0.73
POLQ	chr3q13.33	-0.73
BRIP1	chr17q22.2	-0.74
MAVS	chr20p13	-0.75
CDCA2	chr8p21.2	-0.76
GTF3C3	chr2q33.1	-0.76
RABEPK	chr9q33.3	-0.77
PSMF1	chr20p13	-0.78
GK5	chr3q23	-0.79
NUP160	chr11p11.2	-0.79
SNORD104	chr17q23.3	-0.79
RBCK1	chr20p13	-0.79
TRAF3IP3	chr1q32.3-q41	-0.79
TMEM19	chr12q21.1	-0.79
KRTAP7-1	chr21q22.1	-0.79
MCM10	chr10p13	-0.80
PARP9	chr3q21	-0.82
NUF2	chr1q23.3	-0.83
DNA2	chr10q21.3-q22.1	-0.84
GIPC1	chr19p13.1	-0.86
ITSN1	chr21q22.1-q22.2	-0.86
CDC7	chr1p22	-0.86
MYO9B	chr19p13.1	-0.87
CYP27B1	chr12q13.1-q13.3	-0.88
ZNF655	chr7q22.1	-0.88
H2AFY	chr5q31.3-q32	-0.90
MYH8	chr17p13.1	-0.90
HECTD1	chr14q12	-0.90
OAF	chr11q23.3	-0.91
SLFN13	chr17q12	-0.92
ANKS6	chr9q22.33	-0.92

TRIM62	chr1p35.1	-0.92
DLEU2	chr13q14.3	-0.93
OVOL2	chr20pter-q11.23	-0.94
SP100	chr2q37.1	-0.96
SREBF1	chr17p11.2	-0.99
MCM8	chr20p12.3	-0.99
INTS6	chr13q14.3	-1.00
GALK1	chr17q24	-1.00
ZNF83	chr19q13.3	-1.03
SH3D20	chr17q21.31	-1.04
MRPL20	chr1p36.3-p36.2	-1.05
MAP4K2	chr11q13	-1.06
RAB3IP	chr12q14.3	-1.07
RTKN2	chr10q21.2	-1.09
SNX5	chr20p11	-1.12
SMEK2	chr2p16.1	-1.12
HIST1H2AD / HIST1H3D	chr6p21.3	-1.13
IZUMO2	chr19q13.33	-1.15
LRRFIP1	chr2q37.3	-1.16
KRAS	chr12p12.1	-1.16
OAS3	chr12q24.2	-1.18
LMO1	chr11p15	-1.21
KIF24	chr9p13.3	-1.24
UNC45B	chr17q12	-1.27
TIAM1	chr21q22.1|21q22.11	-1.41
CSTL1	chr20p11.21	-1.42
IL2RA	chr10p15-p14	-1.43
RNF165	chr18q21.1	-1.46
C8B	chr1p32	-1.48
NDST4	chr4q26	-1.52
SLC22A1	chr6q26	-1.61
PCDH7	chr4p15	-1.65
USP2	chr11q23.3	-1.89
HIST1H3G	chr6p21.3	-2.72
