# Supplementary material for: Identification of the IGF1/PI3K/NF κB/ERK gene signalling networks associated with chemotherapy resistance and treatment response in high-grade serous epithelial ovarian cancer
Source: BMC Cancer. 2013 Nov 16;13:549. doi: 10.1186/1471-2407-13-549 (PMC3840597; doi:10.1186/1471-2407-13-549)
Supplement: Additional file 2 — Differentially expressed genes in a resistant cohort compared to a sensitive cohort. The gene list was derived from an independent in silico validation of gene expression analysis using TCGA ovarian cancer data sets (19 sensitive and 25 resistant samples) with identical data analysis parameters as applied for the discovery cohort. [file 1471-2407-13-549-S2.pdf]

Gene Symbol	Chromosomal Location	log2(FoldChange)
ADH1B	chr4q23	3.12
IL21R	chr16p11	2.08
IGLV1-44	chr22q11.2	1.78
IGL	chr22q11.1-q11.2	1.77
IL2RA	chr10p15-p14	1.68
HUNK	chr21q22.1	1.62
MASP1	chr3q27-q28	1.57
BATF	chr14q24.3	1.55
LOC91316	chr22q11.23	1.53
MEOX2	chr7p22.1-p21.3	1.50
KIF25	chr6q27	1.48
TCP10	chr6q27	1.48
TBXA2R	chr19p13.3	1.39
NLRP1	chr17p13.2	1.39
NCF4	chr22q13.1	1.34
ZNF804A	chr2q32.1	1.34
PTGER3	chr1p31.2	1.27
AGTR1	chr3q24	1.22
IL21R	chr16p11	1.22
HS2ST1	chr1p22.3	1.17
ELTD1	chr1p33-p32	1.09
CLDN5	chr22q11.21	1.06
PSD3	chr8p21.3	1.05
KDR	chr4q11-q12	1.03
CEP250	chr20q11.22	1.03
FAM69A	chr1p22.1	1.01
SEMA3G	chr3p21.1	0.99
CCDC88A	chr2p16.1	0.98
VAV1	chr19p13.2	0.93
PCDH17	chr13q21.1	0.92
GLIPR1	chr12q21.2	0.91
MARCKS	chr6q22.2	0.89
MARCKS	chr6q22.2	0.80
MVK	chr12q24	0.80
ZFHX4	chr8q21.11	0.79
AQP1	chr7p14	0.77
COL4A2	chr13q34	0.76
PPAP2A	chr5q11	0.74
RRAGC	chr1p34	0.71
TUBB6	chr18p11.21	0.70
MARCKS	chr6q22.2	0.69
TULP4	chr6q25-q26	0.68

EIF2C3	chr1p34.3	0.67
ATPAF2	chr17p11.2	0.67
TRAF3	chr14q32.32	0.63
SLC38A7	chr16q21	0.61
ENPP2	chr8q24.1	0.60
STX7	chr6q23.1	0.58
FNDC3B	chr3q26.31	0.53
WDR1	chr4p16.1	0.52
HEXB	chr5q13	0.50
GNG11	chr7q21	0.50
ACTR2	chr2p14	0.48
TXLNA	chr1p35.1	0.47
CAP1	chr1p34.2	0.44
RTN4	chr2p16.3	0.31
SEPT2nd	chr2q37	-0.30
HADHB	chr2p23	-0.34
RPL7	chr8q21.11	-0.36
GAPDH	chr12p13	-0.38
ZNF79	chr9q34	-0.39
ANP32B	chr9q22.32	-0.47
HPS6	chr10q24.32	-0.50
NCL	chr2q37.1	-0.54
NCKIPSD	chr3p21	-0.55
TTC4	chr1p32.3	-0.55
PRNP	chr20p13	-0.58
NDUFA10	chr2q37.3	-0.62
EEF1B2	chr2q33.3	-0.62
PER2	chr2q37.3	-0.62
UQCRC2	chr16p12	-0.67
MIPEP	chr13q12	-0.67
AVPI1	chr10q24.2	-0.69
PDCD4	chr10q24	-0.71
PRPS2	chrXp22.3-p22.2	-0.73
DPY19L4	chr8q22.1	-0.75
FOXO4	chrXq13.1	-0.78
SHANK2	chr11q13.2	-0.79
FES	chr15q26.1	-0.80
SOX17	chr8q11.23	-0.85
ZNF239	chr10q11.22-q11.23	-0.88
GPM6B	chrXp22.2	-0.92
DDX11	chr12p11	-1.00
TSNAXIP1	chr16q22.1	-1.01
RAGE	chr14q32	-1.08

IGF1R	chr15q26.3	-1.10
CARD10	chr22q13.1	-1.13
HEMK1	chr3p21.3	-1.14
FKBP1B /// MFSD2B	chr2p23.3	-1.15
ZZEF1	chr17p13.2	-1.20
NPR2	chr9p21-p12	-1.21
MATN2	chr8q22	-1.22
LRIG1	chr3p14	-1.25
CTAGE11P	chr13q22.2	-1.33
OAZ3	chr1q21.3	-1.38
DEPDC6	chr8q24.12	-1.40
FBXL18	chr7p22.2	-1.44
FGFR2	chr10q26	-1.46
SMO	chr7q32.3	-1.47
PLCE1	chr10q23	-1.52
SNRNP200	chr2q11.2	-1.53
GCKR	chr2p23	-1.55
CD200	chr3q12-q13	-1.73
CHL1	chr3p26.1	-1.79
CD200	chr3q12-q13	-1.82
EYA2	chr20q13.1	-1.83
BMP8A	chr1p34.3	-1.91
DLEC1	chr3p21.3	-2.43
MAPRE3	chr2p23.3-p23.1	-2.70
